# Genome-wide expression profile of the response to spinal cord injury in *Xenopus laevis* reveals extensive differences between regenerative and non-regenerative stages

**DOI:** 10.1186/1749-8104-9-12

**Published:** 2014-05-22

**Authors:** Dasfne Lee-Liu, Mauricio Moreno, Leonardo I Almonacid, Víctor S Tapia, Rosana Muñoz, Javier von Marées, Marcia Gaete, Francisco Melo, Juan Larraín

**Affiliations:** 1 Center for Aging and Regeneration, Millennium Nucleus for Regenerative Biology, Departamento de Biología Celular y Molecular, Facultad de Ciencias Biológicas, Pontificia Universidad Católica de Chile, Alameda 340, Santiago, Chile; 2 Departamento de Genética Molecular y Microbiología, Facultad de Ciencias Biológicas, Pontificia Universidad Católica de Chile, Alameda 340, Santiago, Chile

**Keywords:** Cell cycle, *HOX* genes, Immune system, Inflammation, Metabolism, Neurogenesis, Regenerative organisms, RNA-Seq, Spinal cord regeneration, *Xenopus laevis*

## Abstract

**Background:**

*Xenopus laevis* has regenerative and non-regenerative stages. As a tadpole, it is fully capable of functional recovery after a spinal cord injury, while its juvenile form (froglet) loses this capability during metamorphosis. We envision that comparative studies between regenerative and non-regenerative stages in *Xenopus* could aid in understanding why spinal cord regeneration fails in human beings.

**Results:**

To identify the mechanisms that allow the tadpole to regenerate and inhibit regeneration in the froglet, we obtained a transcriptome-wide profile of the response to spinal cord injury in *Xenopus* regenerative and non-regenerative stages. We found extensive transcriptome changes in regenerative tadpoles at 1 day after injury, while this was only observed by 6 days after injury in non-regenerative froglets. In addition, when comparing both stages, we found that they deployed a very different repertoire of transcripts, with more than 80% of them regulated in only one stage, including previously unannotated transcripts. This was supported by gene ontology enrichment analysis and validated by RT-qPCR, which showed that transcripts involved in metabolism, response to stress, cell cycle, development, immune response and inflammation, neurogenesis, and axonal regeneration were regulated differentially between regenerative and non-regenerative stages.

**Conclusions:**

We identified differences in the timing of the transcriptional response and in the inventory of regulated transcripts and biological processes activated in response to spinal cord injury when comparing regenerative and non-regenerative stages. These genes and biological processes provide an entry point to understand why regeneration fails in mammals. Furthermore, our results introduce *Xenopus laevis* as a genetic model organism to study spinal cord regeneration.

## Background

Species across the animal kingdom show a variable range of regenerative ability, with some fish and amphibians capable of regenerating complete appendages after amputation during their entire lifespan, while mammals in general lack this capacity. This remarkable ability shown by regenerative animals is of great interest, as was elegantly described by LV Polezhaev: ‘In order to study why regeneration of organs does not occur in those animals which do not possess regenerative capacity, it is necessary to know how the process of regeneration occurs in animals which do possess regenerative capacity’ [1]. While mammals are unable to regenerate during most stages of their lifetime, they usually possess a higher regenerative capacity during early embryonic development [2,3]. In fact, young children are capable of fingertip regeneration after accidental complete amputation, as long as the amputation level does not go further than the first phalange and no sutures or invasive treatments are used [4]. It is worth noting that ‘regenerative ability of lost body parts’ [5] is, to some extent, shared by all species, with some of them maintaining it throughout their lifespan (for example, fish and salamander), while others lose it progressively during development (for example, frogs and human beings) [6,7]. The progressive loss of the regenerative ability provides a powerful experimental tool for comparing regenerative with non-regenerative stages within the same species.

A medical problem that can be studied through comparative studies between regenerative and non-regenerative animals is spinal cord injury and regeneration. Human beings and mammals suffer from irreversible damage after a spinal cord injury that leads to paralysis (loss of motor function), and impairment of sensory and autonomic function below the injury site, strongly altering quality of life and with a substantial cost to society [8,9]. Spinal cord injury is commonly caused by trauma, where damaged vertebrae compress or transect the spinal cord tissue, causing ‘primary damage’, resulting in hemorrhage and death of neurons and glia. This is followed by ‘secondary damage’, which results from the recruitment of inflammatory cells and reactive astrocytes and the formation of a glial scar that becomes a physical barrier to axonal regeneration [8,10]. In addition, intrinsic and extrinsic factors inhibit axon growth and neurogenesis in mammals after spinal cord injury, contributing to its irreversibility [11-13]. Several therapies tested in clinical settings, including stem cell transplantation trials [9], have had only limited effects on functional recovery, indicating that further understanding of the basic mechanisms underlying spinal cord regeneration is required [14].

Unlike mammals, teleost fish and amphibians like adult urodeles (for example, newts) and anuran larvae (for example, *Xenopus*) are capable of functional recovery after spinal cord transection [6,7,12,15,16]. In *Xenopus*, regeneration is restricted to larvae or tadpole stages (stages 50 to 54, R-stages), while once metamorphosis has concluded, the resulting froglets are unable to regenerate (stages 58 to 66, NR-stages) [17-21]. The experimental paradigm provided by *Xenopus* enables study of the mechanisms required for regeneration that are missing in non-regenerative organisms, and provides a model to test for gain-of-function treatments that could enhance regeneration. However, a comparison at the transcriptomic level of the response to spinal cord injury in regenerative (R-) and non-regenerative (NR-) stages in *Xenopus* has not been performed. We envision that characterizing this model at a high-throughput level can lead to the identification of mechanisms, signaling pathways, gene networks, and factors that either promote or inhibit spinal cord regeneration.

The arrival of large-scale sequencing technologies has enabled the study of regeneration at the transcriptome level in diverse organisms, such as flatworms [22-24], cricket [25], sheep [26], and in such tissues as the lens [27], liver [28] and deer antlers [29]. In *Xenopus laevis*, there have been no previous reports using large-scale sequencing to study spinal cord regeneration, although the Amaya group published a paper that used microarrays on tail regeneration in *Xenopus tropicalis*[30], from which it was possible to identify the requirement of reactive oxygen species for successful tail regeneration [31].

Here, we aimed to identify differences in the transcriptional landscape deployed in response to spinal cord transection in R- and NR- stages in *Xenopus laevis*. Spinal cords from animals at 1, 2 and 6 days post transection (dpt) or after sham operation were dissected and high-throughput RNA sequencing was performed. Changes in response to spinal cord injury were studied, comparing transected against sham-operated animals at the time points indicated, and by further comparing the responses between R- and NR-stages. We found that these two stages regulated a very different set of transcripts in response to injury, and that extensive transcriptome changes in regenerative tadpoles were already observed at 1 dpt, while non-regenerative froglets displayed the highest levels of transcriptional regulation at 6 dpt. This indicated very different kinetics in the responses shown by these two stages. Furthermore, a search for processes involved in spinal cord regeneration showed that genes related to neurogenesis and the axonal growth cone were differentially regulated, allowing us to identify specific proneural factors exclusively regulated in the R-stage and several growth cone genes that were only downregulated in the NR-stage. In addition, gene ontology enrichment analysis showed widespread differences in the response to injury of genes from biological processes, such as cell cycle, response to stress, metabolism, development, and immune response and inflammation. We have validated the differential expression of several genes involved in these processes using low-scale validation (RT-qPCR). We have also identified previously uncharacterized transcripts differentially regulated after spinal cord injury, providing a subset of genes that can open unexpected avenues to the understanding of spinal cord regeneration. In summary, we have found extensive differences in the timing and composition of the transcriptome deployed in response to spinal cord injury in regenerative and non-regenerative *Xenopus laevis*. We envision that the set of genes and biological processes identified here represent a starting point for the study of their function and how their modulation can improve spinal cord regeneration in animals that lack this capability.

## Results

### Response to spinal cord transection in *Xenopus laevis* regenerative and non-regenerative stages

**Figure 1:**
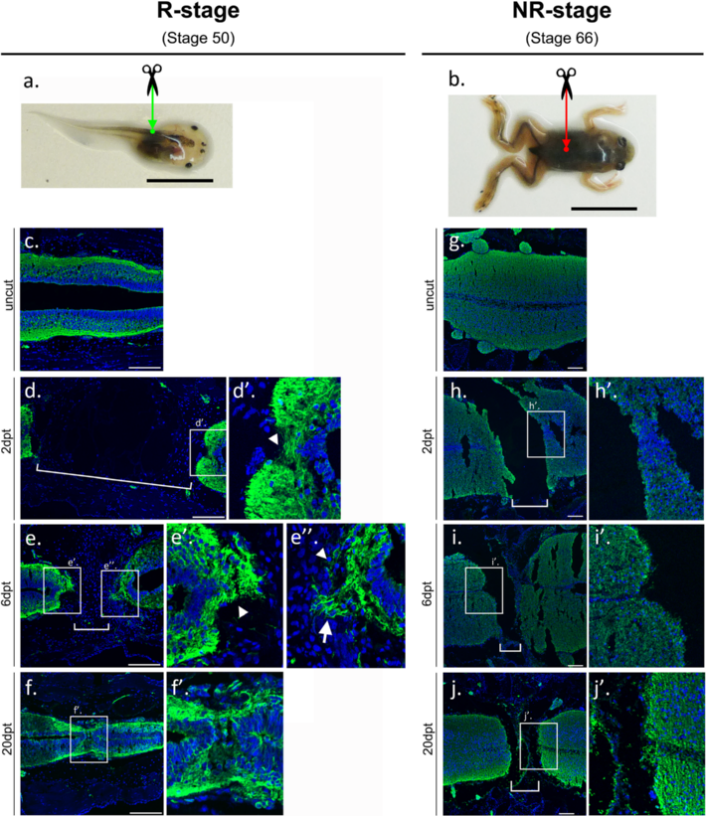
**Spinal cord transection and regeneration in *Xenopus laevis *regenerative and non-regenerative stages. (a,b)** *Xenopus laevis* R-stage **(a)** and NR-stage **(b)**, indicating the transection site at the mid-thoracic point (green and red arrows). **(c-j)** Immunofluorescence in longitudinal spinal cord sections using an anti-acetylated tubulin antibody to stain for axon growth after the injury. Insets show magnifications of boxed areas. While axon growth occurs across the lesion site in the R-stage, this is not the case for the NR-stage. Brackets, ablation gap. Arrowheads, wrapping of axons around the stump. Arrow, wisping or axonal growth tips into the ablation gap. Scale bars: **(a, b)** 1 cm, **(c-j)** 100 μm. dpt, days post transection; NR, non-regenerative; R, regenerative.

To characterize the response to spinal cord injury in regenerative and non-regenerative stages in *Xenopus laevis*, we performed full transection of the spinal cord at the midpoint between fore and hind limbs (or limb buds) in both tadpoles (stage 50; R-stage, Figure 1a) and froglets (stage 66; NR-stage, Figure 1b), and detected axonal tracts using immunofluorescence for acetylated tubulin (Figure 1c-j). Spinal cord transection severed all innervation between the rostral and caudal regions of the spinal cord, leaving an ablation gap between the rostral and caudal stumps, which were fully separated at 2 dpt (Figure 1d,h, see brackets). Interestingly, at 2 dpt in the R-stage, axons had started to wrap around the stumps, something that was not observed in the NR-stage (Figure 1d′ arrowhead, and 1h′).

At 6 dpt in R-stage animals, we observed a higher density of axons wrapped around the stumps (Figure 1e′,e″, arrowheads) and axons starting to extend their tips into the ablation gap (Figure 1e,e″, arrow), a process named ‘wisping’ during salamander spinal cord regeneration [32]. By 20 dpt in the R-stage, axonal tracts crossed the injury site and connection between rostral and caudal stumps was achieved (Figure 1f,f′), although ependymal canal continuity was only completed in some animals (not shown). Conversely, NR-stage animals continued to have an ablation gap at 20 dpt, with no wrapping of the stumps by axons (Figure 1h′-j′), and normal anatomy and histology was never recovered (Figure 1h-j). This anatomical and histological recovery had functional correlation with our previous work [21]. Indeed, R-stage animals gradually recover after the injury; reaching full recovery at 30 dpt. Contrary to that, no recovery is observed in NR-stage animals [21].

These results show that R- and NR-stage animals have a completely different response to spinal cord injury and regenerative capacity, and that differences can already be observed within the first few days after injury. This characterization of the response to transection allowed us to select early response time points for further study.

### Genome-wide expression profile in response to spinal cord transection

Although anatomical and functional recovery in the R-stage is achieved 3 to 4 weeks after transection [21], clear histological differences were already observed within the first 2 days after injury between the responses in R- and NR-stages. Thus, we aimed to identify these early response genes and biological processes using high-throughput mRNA sequencing (RNA-Seq), comparing R- and NR-stages at 1, 2 and 6 dpt. Spinal cords of transected R- and NR-stage animals were dissected by isolating a fragment caudal to the lesion site. Equivalent samples were obtained from sham-operated animals to which only an incision to the dorsal skin and muscle was performed, leaving the spinal cord uninjured. These were used as controls, allowing a normalization baseline that excluded changes occurring as a result of damage to tissues other than the spinal cord. For technical reasons, we decided to isolate a fragment of the spinal cord caudal to the transection site, because in stage 50 animals the rostral segment between the transection site and the hindbrain is very short. While this implied a loss of information of some regenerating axons in the rostral segment, axonal growth from the caudal segment (Figure 1e) plus the presence of ependymal cells that could be giving rise to new neurons made our sample of great interest.

To determine the abundance of each detected transcripts, reads were mapped using the UniGene transcript database as a reference (*Xenopus laevis* UniGene build #90), which contained 35,669 transcripts. The genome was not used, owing to its current draft status. The sequencing quality (over 90% Q30 or higher after trimming), the percentage of mapped reads (on average, 27%) and the number of detected transcripts (26,000 to 27,000) were comparable between samples (Additional file 1), supporting the robustness of the sequencing results.

**Figure 2:**
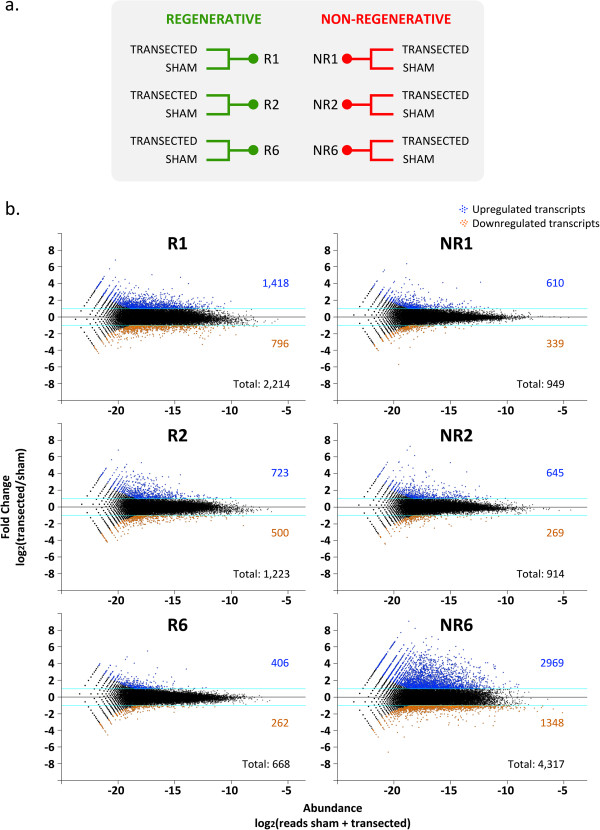
**Differentially expressed transcripts in response to spinal cord transection. (a)** Samples were isolated after 1, 2, or 6 days post-surgery (transection or sham) from regenerative and non-regenerative animals. **(b)** *MA* plots depicting differentially expressed transcripts. Blue dots and numbers correspond to transcripts upregulated after transection and orange dots and numbers to those downregulated after transection.

To identify differentially expressed transcripts, we performed a pairwise normalization of each transected sample and generated six lists (R1, R2, R6, NR1, NR2, and NR6), which contained all transcripts that significantly changed in response to spinal cord injury (Figure 2a; see Additional file 2 for full transcript list). The following parameters were used to define significant differential expression between transected and sham-operated animals: (i) fold change, FC ≥ 2 for upregulated transcripts and FC ≤ 0.5 for downregulated transcripts, (ii) a minimum abundance of reads detected in transected + sham > 10, and (iii) *P* < 0.01. Based on these criteria, we found 7,431 transcripts with differential expression in the six time points studied.

To visualize the transcriptome’s response to injury, *MA* plots depicting the fold change on the *y*-axis and transcript abundance on the *x*-axis were constructed (Figure 2b). Transcripts with a significant differential expression are shown as blue (upregulated transcripts after transection) or orange (downregulated transcripts after transection) dots (Figure 2b). Interestingly, the number of differentially regulated transcripts in the R-stage was higher on days 1 and 2 than on day 6 (Figure 2b, graphs on left). Conversely, in the NR-stage, this number was lower on days 1 and 2, and increased by day 6 (Figure 2b, graphs on right). More transcripts were therefore regulated in response to spinal cord injury at early time points in the R-stage than in the NR-stage and a clear difference in the timing of the response was detected.

### Identification of transcripts that respond differentially to spinal cord injury in regenerative and non-regenerative stages

**Figure 3:**
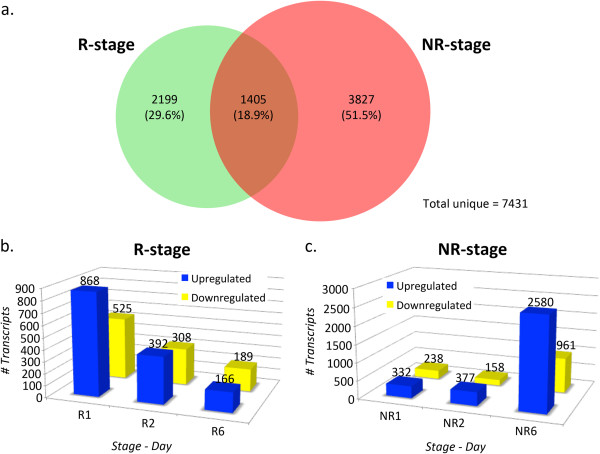
**Comparison of the response to spinal cord injury in regenerative and non-regenerative stages. (a)** Venn diagram showing all differentially expressed transcripts detected in all time points in R- (green) and NR-stage animals (red). Out of a total of 7,431 transcripts detected in all samples as differentially expressed, 18.9% were differentially regulated in both stages, 29.6% regulated exclusively in the R-stage and 51.5% in the NR-stage. **(b, c)** Total transcripts that responded differently in R- **(b)** and NR-stages **(c)** are depicted as bar graphs. Blue bars, upregulated transcripts; yellow bars, downregulated transcripts.

The fact that our model organism provides R- and NR-stages allows our analysis to go one step further, enabling us to identify transcripts that respond differentially to spinal cord injury in R- and NR-stages. This unique possibility favors the identification of transcripts and biological processes required for spinal cord regeneration, as well as those that inhibit regeneration. First we compared all differentially expressed transcripts from the six lists generated (R1, R2, R6, NR1, NR2, and NR6) to determine whether they activated the same set of transcripts in response to transection. We found that 18.9% transcripts were regulated in both the R- and the NR-stage, including those with an opposite regulation when comparing one stage with the other (95 out of 1405 transcripts). The remaining 81.1% were only regulated in the R- (29.6%) or the NR-stage (51.5%) (Figure 3a). These results highlight the differences in the transcript repertoire deployed in response to injury in R- and NR-stages.

To identify transcripts that responded differently in R- and NR-stages amongst the list of differentially expressed transcripts, we performed a second pairwise comparison for each time-point (R1 versus NR1; R2 versus NR2, and R6 versus NR6) and calculated an FC ratio (FC of a given transcript in one stage, divided by the FC of the same transcript in the other stage). We considered a different response in R- and NR-stages when the FC ratio was ≥ 2 or ≤ 0.5. This yielded a total of 5,767 genes that responded differentially to spinal cord injury in both stages (Figure 3b, c; see Additional file 3 for full transcript list). As expected from the analysis of the lists containing differentially expressed transcripts (Figure 2b), the number of transcripts that responded differently was higher on day 1 in the R-stage than in the NR-stage, and in the R-stage decreased rapidly towards day 6, while exactly the opposite was observed in the NR-stage transcriptome (Figure 3b, c).

To identify transcripts that showed the highest differences in their regulation between R- and NR-stages, they were ordered using the ‘FC ratio’ to select top differentially regulated transcripts, and then further filtered to select the most abundantly expressed transcripts (Tables 1, 2, 3; see Methods for filtering criteria). The top regulated transcripts were highly represented by: (i) genes involved in metabolic processes, at 1 and 2 days after injury (*serine dehydratase*, two isoforms of *glutamate-ammonia ligase*, *betaine-homocysteine S- methyltransferase, uncoupling protein 2*), and two transcripts with high sequence similarity to known transcripts from other species, for example, *hypothetical protein MGC78829* with 95.1% identity to the *Xenopus tropicalis uncoupling protein 2*, and *hypothetical protein LOC100036942* with 96.1% identity to the *X. tropicalis l-serine dehydratase/L-threonine deaminase* (Tables 1, 2); (ii) genes involved in cell cycle processes at 6 days after injury, including *ribonucleotide reductase M2 B*, *Karyopherin alpha 2, Karyopherin alpha-2 subunit like*, *MCM3 minichromosome maintenance deficient 3* and the previously uncharacterized *hypothetical protein MGC81499* with a 97.6% identity to the *Xenopus tropicalis cyclin-dependent kinase 2* (Table 3).

**Table 1:** Top 10 differentially regulated transcripts 1 day post transection

**GenBank ID**	**Gene name**	**UniGene cluster description**	**log**_**2**_**FC**		**FC ratio**
			**R1**	**NR1**	
BC070531	*MGC78829*	*Hypothetical protein MGC78829*	-1.06	4.15	36.92
BC129680	*sds*	*Serine dehydratase*	-3.27	1.92	36.51
BC076749	*stc1*	*Stanniocalcin 1*	5.07	0.31	27.06
BC129695	*LOC100036942*	*Hypothetical protein LOC100036942*	-2.20	1.63	14.18
NM_001092398	*glul-a*	*Glutamate-ammonia ligase*	-2.28	1.51	13.76
BC073470	*glul-b*	*Glutamate-ammonia ligase*	-1.98	1.61	12.09
BC070543	*aqp3-a*	*Aquaporin 3 (Gill blood group)*	3.99	0.49	11.29
BC084414	*bhmt*	*Betaine-homocysteine S-methyltransferase*	-0.98	2.37	10.20
BC041489	Unknown	*Xenopus laevis, clone IMAGE:4930284, mRNA*	-1.48	1.26	6.70
BC044682	*ucp2*	*Uncoupling protein 2 (mitochondrial, proton carrier)*	-0.80	1.94	6.69

Top 10 transcripts that, in addition to being differentially expressed after injury, show a different response when comparing regenerative and non-regenerative stages. Selection criteria: *P* < 0.01; sum of reads (transected + sham) higher or equal to the mean value of all transcripts. FC ratio: ratio between FC(R)/FC(NR) or FC(NR)/FC(R) (see Figure 2b for selection criteria). FC, fold change (transected/sham).

**Table 2:** Top 10 differentially regulated transcripts 2 days post transection

**GenBank ID**	**Gene name**	**UniGene cluster description**	**log**_**2**_**FC**		**FC ratio**
			**R2**	**NR2**	
BC078115	*hsp70*	*Heat shock 70 kDa protein*	-0.65	4.22	29.26
BC070531	*MGC78829*	*Hypothetical protein MGC78829*	-2.22	2.51	26.66
BC068675	*cnfn-a*	*Cornifelin*	-1.81	2.85	25.19
BC141766	*LOC100049771*	*Hypothetical protein LOC100049771*	1.39	5.32	15.26
BC157718	*cfos-A*	*C-fos proto-oncogene*	-1.88	1.73	12.19
BC129680	*sds*	*Serine dehydratase*	-1.72	1.73	10.93
BC084644	*hla-dqa1*	*Major histocompatibility complex, class II, DQ alpha 1*	-2.52	0.68	9.21
BC167488	*LOC495461*	*Hypothetical LOC495461*	-2.01	1.16	8.99
BC044682	*ucp2*	*Uncoupling protein 2 (mitochondrial, proton carrier)*	-1.00	2.07	8.44
AB075925	*olfm4*	*Olfactomedin 4*	-0.35	2.32	6.36

Top 10 transcripts that, in addition to being differentially expressed after injury, show a different response when comparing regenerative and non-regenerative stages. Selection criteria: *P* < 0.01; sum of reads (transected + sham) higher or equal to the mean value of all transcripts. FC ratio: ratio between FC(R)/FC(NR) or FC(NR)/FC(R) (see Figure 2b for selection criteria). FC, fold change (transected/sham).

**Table 3:** Top 10 differentially regulated genes 6 days post transection

**GenBank ID**	**Gene name**	**UniGene cluster description**	**log**_**2**_**FC**		**FC ratio**
			**R6**	**NR6**	
BC041209	*rrm2b*	*Ribonucleotide reductase M2 B (TP53 inducible)*	-0.29	5.76	66.26
X82012	*kif4a*	*Kinesin family member 4A*	-0.24	5.68	60.55
BC043778	*kpna2*	*Karyopherin alpha 2 (RAG cohort 1, importin alpha 1)*	-0.40	4.75	35.51
BC060435	*MGC68771*	*Hypothetical protein MGC68771*	1.02	6.08	33.36
BC070640	*MGC81499*	*Hypothetical protein MGC81499*	-0.41	4.58	31.78
AJ557446	*kpna2*	*Karyopherin alpha-2 subunit like*	-0.37	4.35	26.35
BC084431	*kifc1*	*Kinesin family member C1*	-0.18	4.39	23.75
BC154984	*ctsk*	*Cathepsin K*	0.69	5.17	22.32
BC099253	*LOC446922*	*Hypothetical protein LOC446922*	-0.84	3.58	21.41
BC044051	*mcm3*	*MCM3 minichromosome maintenance deficient 3 (S. cerevisiae)*	-0.23	4.18	21.26

Top 10 transcripts that, in addition to being differentially expressed after injury, show a different response when comparing regenerative and non-regenerative stages. Selection criteria: *P* < 0.01; sum of reads (transected + sham) higher or equal to the mean value of all transcripts. FC ratio: ratio between FC(R)/FC(NR) or FC(NR)/FC(R) (see Figure 2b for selection criteria). FC, fold change (transected/sham).

In summary, R- and NR-stages regulate very different sets of transcripts after spinal cord injury, and we were able to select those that showed a different response when comparing both stages. This set of transcripts reinforced the different timing detected previously. In addition, it is very valuable, as it contains candidates that could explain the difference in regenerative ability between R- and NR-stages; in particular, the top differentially regulated transcripts were obtained, as they are possible candidates for future *in vivo* functional studies.

### Genes related to neurogenesis and axonal regeneration were differentially regulated after spinal cord injury in regenerative and non-regenerative stages

Having obtained a set of transcripts that responded differentially to spinal cord injury in R- and NR-stages, we sought to determine which gene ontologies (GOs) they belonged to, searching for biological process, molecular function, and cellular component. We began by searching transcripts belonging to biological processes known to be involved in spinal cord regeneration, such as neurogenesis and axonal growth. We have highlighted two groups of particular interest. The first was ‘neurogenesis’ [GO: 0022008], and its associated terms ‘positive regulation of neurogenesis’ [GO: 0050769] and ‘negative regulation of neurogenesis’ [GO: 0050768]. The second was the cellular compartment ‘growth cone’ [GO: 0030426], and its associated terms ‘axonal growth cone’ [GO: 0044295] and ‘growth cone membrane’ [GO: 0032584].

**Figure 4:**
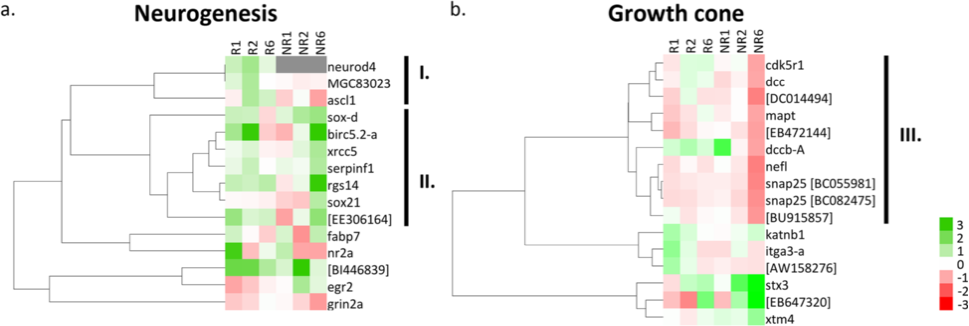
**Differential regulation of genes related to neurogenesis and the axonal growth cone. (a)** Heat map and clustering analysis of differential expression of genes related to neurogenesis, showing one cluster of genes exclusively upregulated in the R-stage (I) and another that shows an early upregulation or no change in the R-stage and a delayed upregulation in the NR-stage (II). **(b)** Heat map and clustering analysis of differentially expressed genes expressed in the axonal growth cone, showing downregulation of a large cluster of genes in the NR-stage 6 days after injury (III). GenBank IDs are shown in square brackets.

Heat maps and clustering analysis of all transcripts associated with these two groups were performed (Figure 4). For neurogenesis related transcripts, we found two main groups. The first included three transcripts that were exclusively upregulated in the R-stage but were absent or downregulated in the NR-stage (Figure 4a, group I), including *neurod4, ascl1*, and *MGC83023* (97.3% sequence identity to the *Xenopus tropicalis achaete-scute homolog-1 like*) [GenBank: BC073667]. In the second group, transcripts were mainly upregulated or unchanged in R1 and R2 (days 1 and 2) and their upregulation was delayed in NR-stages and only observed at 6 dpt (Figure 4a, group II). This group included SOX factors *soxd* and *sox21*. Therefore, amongst transcripts involved in neurogenesis, most were rapidly upregulated in the R-stage and no change or a delayed upregulation was observed in NR-stages, supporting that a proper and timely activation of neurogenesis is required for spinal cord regeneration.

Regarding growth-cone-related transcripts, clustering analysis grouped a large set of transcripts that showed a strong downregulation in NR6, while remaining mainly unchanged or downregulated at a lower magnitude in the R-stage (Figure 4b, group III). Amongst these, we found several genes of interest, including *cdk5r1*, the p35 neuron specific activator of CDK5 required for neurite outgrowth [33], the microtubule-associated tau protein *mapt* and two transcripts for synaptosomal-associated protein *snap25*.

To complement the ontology-annotated lists, we performed a manual search for genes previously published as associated with axonal regeneration [34,35] and found a few more that were not listed in the neurogenesis and growth cone heat maps. We found molecules involved in axonal guidance, including *EPH receptors*, *semaphorins*, the *nerve growth factor receptor ngfr(p75)* and the extracellular matrix serine protease *reelin* involved in neuronal migration (Table 4). We also found a group of genes associated with intrinsic factors known to be involved in axonal regeneration, including *krüppel-like factors* and members of the JAK-STAT signaling pathway (Table 4). In summary, several genes associated with neurogenesis and axonal regeneration, including molecules expressed in the growth cone and axonal guidance genes, were differentially regulated after injury when comparing R- and NR-stages.

**Table 4:** Manual search for genes associated with axonal regeneration processes show that they are differentially expressed in regenerative and non-regenerative stages

**Category**	**GenBank ID**	**Gene name**	**UniGene cluster description**	**log**_**2**_**FC**					
					**R1**	**R2**	**R6**	**NR1**	**NR2**	**NR6**
Axonal guidance	BC108561	*ngfr*	*Nerve growth factor receptor*	0.21	-0.20	-0.31	-0.16	-0.05	1.20
	X91191	*epha4-a*	*EPH receptor A4*	-0.25	-0.09	0.25	-0.42	-0.48	-1.36
	BC043626	*epha4-b*	*EPH receptor A4*	-0.17	0.13	0.52	-0.51	-0.16	-1.28
	BC060745	*epha2*	*EPH receptor A2*	-0.42	0.01	-0.01	0.07	1.01	1.46
	BC077964	*sema4b*	*Sema domain, immunoglobulin domain (Ig), transmembrane domain and short cytoplasmic domain, (semaphorin) 4B*	-0.35	-0.01	0.19	0.08	0.43	-1.00
	BC124869	*sema3f*	*Sema domain, immunoglobulin domain (Ig), short basic domain, secreted, (semaphorin) 3 F*	0.19	0.30	0.18	0.29	1.15	1.64
	AF427525	*reln*	*Reelin*	-0.30	0.21	0.32	-0.52	0.26	-1.16
Intrinsic factors	AY116304	*klf15*	*Krüppel-like factor 15*	-1.49	-0.58	-0.43	-0.46	0.64	-0.84
	BC092147	*klf10*	*Krüppel-like factor 10*	-0.18	-0.10	-0.05	0.28	0.74	1.72
	BC054214	*socs3*	*Suppressor of cytokine signaling 3*	2.12	2.00	1.72	2.15	3.72	4.62
	BC068752	*socs3*	*Suppressor of cytokine signaling 3*	3.31	2.05	1.01	3.40	4.45	6.55
	BC044717	*stat3.2*	*Signal transducer and activator of transcription 3 (acute-phase response factor), gene 2*	0.74	0.61	0.19	0.55	0.98	2.10
	BC086272	*stat6*	*Signal transducer and activator of transcription 6, interleukin-4 induced*	-0.43	0.72	0.47	0.38	0.57	1.62

Two categories are shown: genes related to axonal guidance and genes associated with intrinsic factors involved in axonal regeneration. FC, fold change (transected/sham).

### Global profile of biological processes differentially regulated after spinal cord injury in regenerative and non-regenerative stages

**Figure 5:**
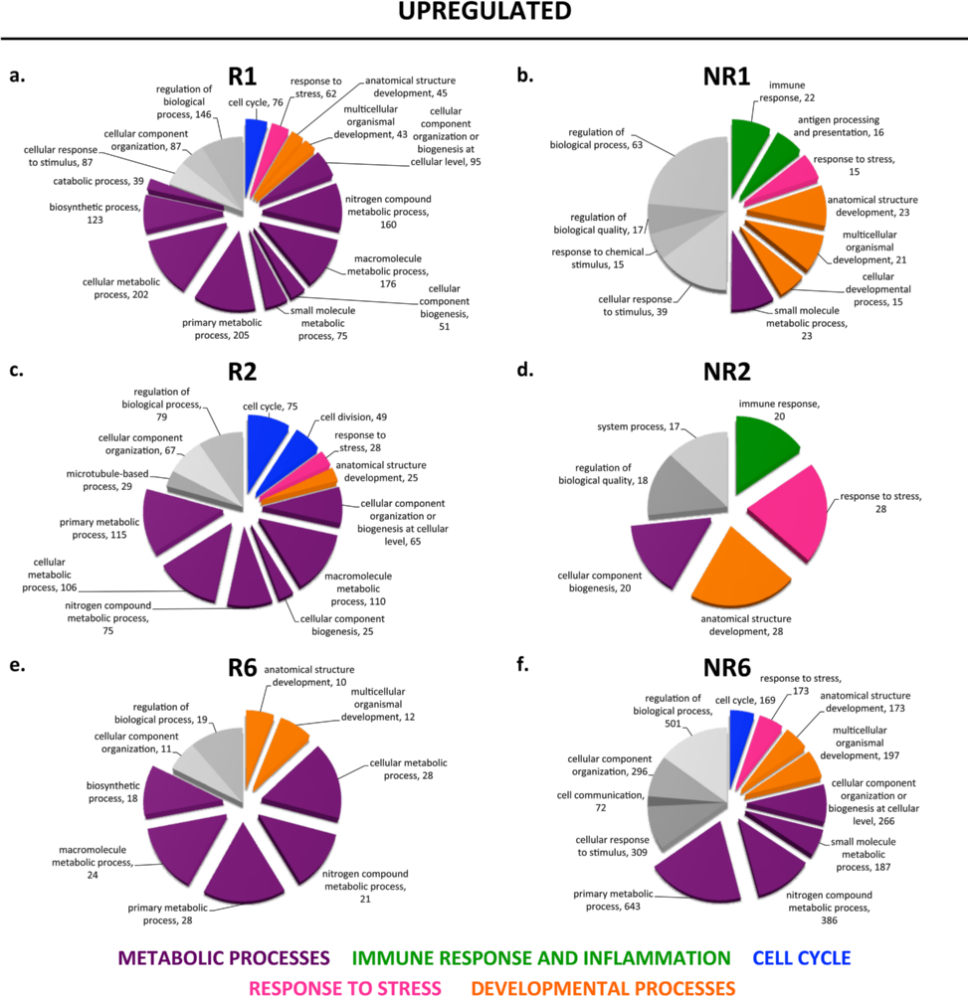
**Gene ontology enrichment analysis for upregulated transcripts.** Gene ontology (GO) enrichment analysis was performed for transcripts that showed a different response to injury when comparing regenerative and non-regenerative stages for Day 1 **(a, b)**, Day 2 **(c, d)**, and Day 6 **(e, f)**. Colors classify GO terms into the following categories: cell cycle, blue; response to stress, pink; developmental processes, orange; metabolic processes, purple; immune response and inflammation, green; others, grey.

**Figure 6:**
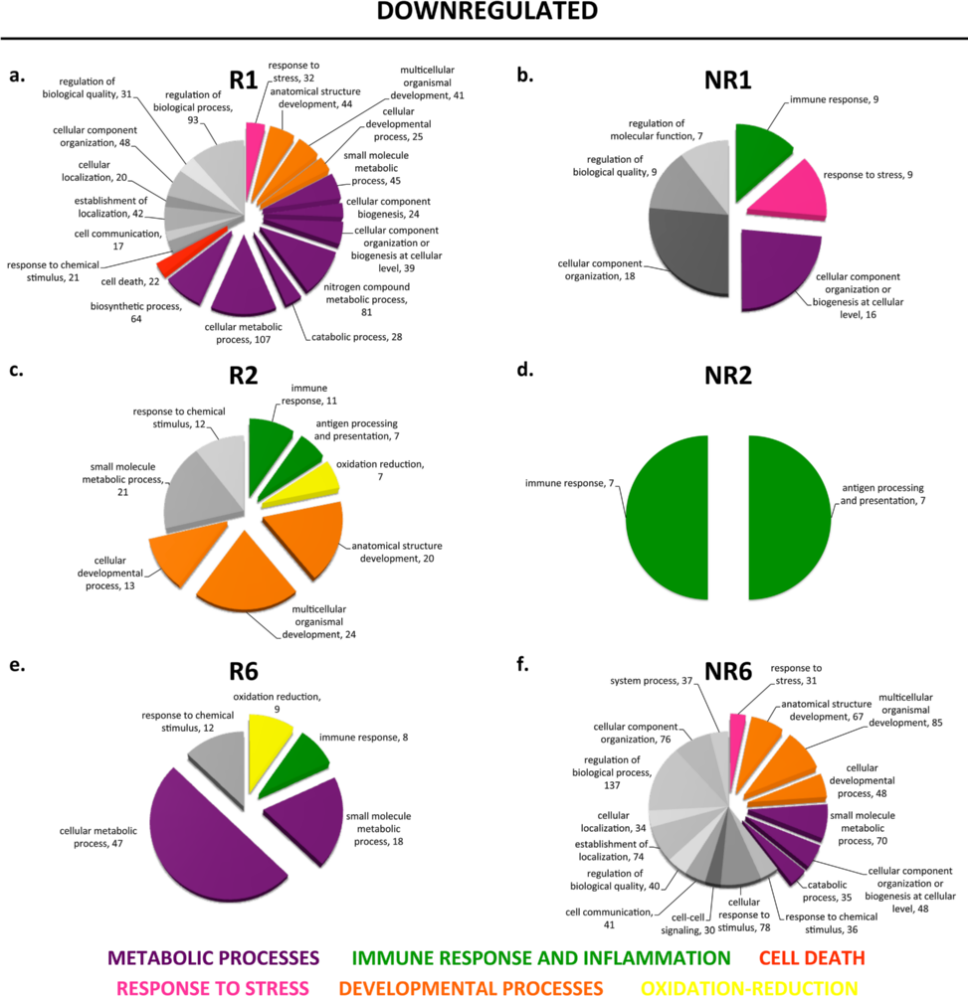
**Gene ontology enrichment analysis for downregulated transcripts.** Gene ontology (GO) enrichment analysis was performed for transcripts that showed a different response to injury when comparing regenerative and non-regenerative stages for Day 1 **(a, b)**, Day 2 **(c, d)**, and Day 6 **(e, f)**. Colors classify GO terms into the following categories: cell cycle, blue; response to stress, pink; developmental processes, orange; metabolic processes, purple; immune response and inflammation, green; others, grey.

**Figure 7:**
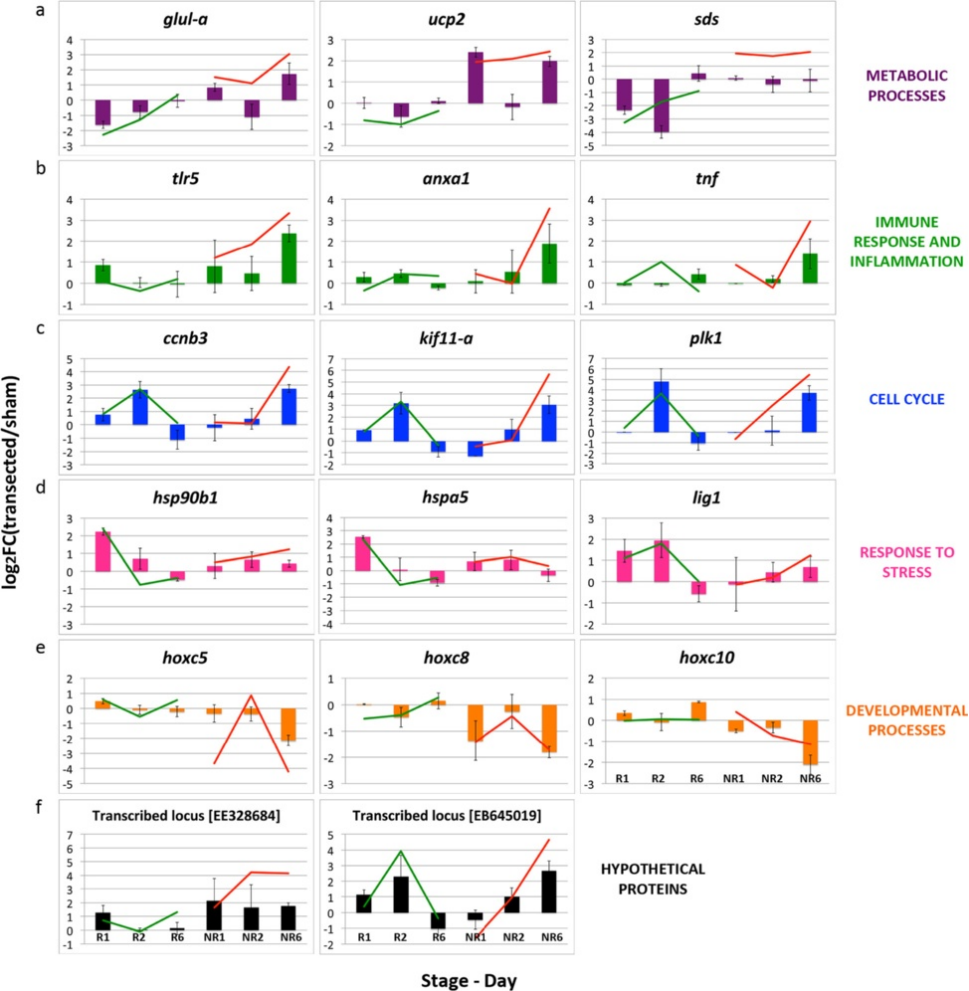
**RT-qPCR data validation for transcripts from gene ontology categories.** Bars show RT-qPCR results for at least two biological replicates prepared from different pools of animals, validating differential expression changes for transcripts from biological processes. **(a)** Metabolic processes. **(b)** Immune response and inflammation. **(c)** Cell cycle. **(d)** Response to stress. **(e)** Developmental processes. **(f)** Hypothetical proteins. Green line, RNA-Seq results for the regenerative stage; red line, RNA-Seq results for the non-regenerative stage. GenBank IDs are shown in square brackets.

This search for biological processes known to be involved in spinal cord regeneration demonstrated that our experimental set-up allows the detection of important differences in response to spinal cord injury in both stages. Consequently, we performed an unbiased GO enrichment analysis to identify the main biological processes regulated in response to injury in R- and NR-stages (Figures 5, 6 and Additional files 3, 4, 5). Importantly, we found that biological processes predominant amongst up- and downregulated transcripts showed very different profiles when comparing R- and NR-stage responses to spinal cord injury (Figures 5, 6). Differential expression of some transcripts from the most relevant gene ontology categories were validated using RT-qPCR in at least two independent biological replicates (Figure 7).

The following general principles emerged from the GO analysis and validation experiments.

#### Metabolism

A high predominance of transcripts involved in metabolic processes was observed in the R-stage. In most regenerative time points, about ten different categories related to metabolic processes represented more than 50% of transcripts that responded differentially to spinal cord injury. Conversely, metabolic processes were not as predominant in NR-stages, particularly during the first 2 dpt (Figures 5, 6; purple). Within these first two days in the NR-stage, only the categories ‘small molecular metabolic process’ and ‘cellular component biogenesis’ were observed, while several other categories were already detected in the R-stage at these time points. Accordingly, many of the genes amongst the top differentially regulated transcripts belonged to these categories (Tables 1, 2). We have experimentally validated the differential expression pattern for three of them: *sds* (*serine dehydratase*), *ucp2* (*uncoupling protein 2*) and *glul-a* (*glutamate-ammonia ligase a*) (Figure 7a). These results suggest a fundamental role for metabolism in allowing a proper response to spinal cord injury.

#### Immune response and inflammation

Categories related to immune response and inflammation were exclusively enriched amongst upregulated transcripts in the NR-stage at 1 and 2 dpt, but were not observed in the R-stage (Figure 5, green). RT-qPCR experiments in biological replicates demonstrated that *tlr5* (*Toll-like receptor 5*), *anxa1* (*Annexin A1*) and *tnf (Tumor necrosis factor)* were only upregulated in the NR-stage, while remaining unchanged in the R-stage (Figure 7b).

#### Cell cycle

Transcripts belonging to the cell cycle categories (‘cell cycle’ and ‘cell division’) were rapidly and transiently upregulated at 1 and 2 dpt in the R-stage, whereas in the NR-stage upregulation of these transcripts was not detected until day 6 (Figure 5, blue). *ccnb3 (cyclin b3)*, *kif11-a (kinesin family member 11 a)* and *plk1 (polo-like kinase 1)* were validated by RT-qPCR, confirming the different temporal activation of this process in R and NR-stages (Figure 7c). It is noteworthy that out of the 209 transcripts identified in the ‘cell cycle’ category, 93 showed an early upregulation in the R-stage and a late upregulation in the NR-stage, demonstrating that a subset of genes was regulated in both stages, but with different timing, while the remaining 106 transcripts were regulated in one stage only. For example, we detected two unannotated genes (that is, transcribed loci), one strongly similar to *cytoplasmic dynein 1 heavy chain 1-like* (GenBank: BX846446) and (GenBank: EG573111), and the other strongly similar to neurogenic transcription factor *neurogenin 1* (GenBank: DC114135), which were exclusively upregulated in the R-stage but not in the NR-stage.

#### Response to stress

The ‘response to stress’ category was transiently enriched in the R-stage at 1 and 2 dpt, while being enriched across all time points in the NR-stage amongst up and downregulated transcripts (Figures 5, 6; pink). Interestingly, out of 281 transcripts detected in this category, only 56 were regulated in both stages and the vast majority were either regulated in the R- or the NR-stage, indicating that the response to stress was different in timing and in the type of genes regulated. Supporting this, we validated the expression pattern of two heat shock proteins *hsp90b1* and *hspa5*, and *lig1* (*Ligase I, DNA, ATP-dependent*), all of which were transiently upregulated in the R-stage only (Figure 7d).

#### Developmental processes

Categories related to developmental processes (‘anatomical structure development’, ‘multicellular organismal development’, ‘cellular developmental process’) showed a very interesting pattern. Although genes corresponding to these categories were upregulated in a similar pattern in R- and NR-stages at all time points, we observed a difference in downregulated genes: these were mainly downregulated during the first two days in the R-stage, something that was only observed at 6 dpt in the NR-stage (Figures 5, 6; orange). It is noteworthy that we detected several *HOX* gene family members, and amongst them we validated expression changes for *hoxc5*, *hoxc8*, *hoxc10*, *hoxd10*, all of which showed a reproducible downregulation in NR6 (Figure 7e and data not shown).

#### Hypothetical proteins

Genes belonging to the ontologies mentioned previously were known and had been characterized for their function in other species. However, the UniGene transcript database also includes transcripts from hypothetical proteins, some of which have been assigned gene ontologies based on their sequence similarity to known genes. We found two examples amongst the top regulated transcripts: *hypothetical protein MGC78829*, which has 95.1% nucleotide sequence identity with the *X. tropicalis uncoupling protein 2* transcript, and *hypothetical protein MGC68771*, with 91.7% nucleotide sequence identity with the *X. tropicalis PREDICTED: heme oxygenase 1-like* transcript. Conversely, we also found transcripts for hypothetical proteins with no previously characterized function. These included two transcribed loci with interesting expression profiles ((GenBank: EE328684) and (GenBank: EB645019)). One exhibited a transient upregulation in the R-stage but sustained regulation in the NR-stage (GenBank: EE328684), similar to response to stress transcripts, while the other (GenBank: EB645019) was upregulated in R2 and NR6, similar to cell cycle transcripts (Figure 7f).

In conclusion, our results showed that transcripts from the aforementioned gene ontologies exhibit differential regulation after spinal cord transection when comparing R- and NR-stages. These changes were reproducible in at least two independent biological replicates, further supporting the robustness of the observed results.

## Discussion

We report the first transcriptome-wide study that compares the response to spinal cord injury in *Xenopus* regenerative (R-) and non-regenerative (NR-) stages. While previous transcriptome studies in spinal cord injury models have been reported, they have only been performed in either mammals with very limited regenerative capabilities [36-43] or in models that regenerate throughout their lifespan, such as the zebrafish and urodele amphibians [44-47], and have used microarrays. Our study, presented here, uses high-throughput sequencing, and provides a unique experimental paradigm, whereby differences in the response to spinal cord injury between these two stages can be identified, which could then explain the difference in regenerative ability.

### Early morphological differences in the response to spinal cord injury between regenerative and non-regenerative stages

We first performed immunofluorescence assays for a comparative characterization of the response to spinal cord transection of these two stages. The presence of R- and NR-stages during *Xenopus* development has previously been characterized. Sims [48] described in 1962 that stage 56 was the latest stage at which animals could survive spinal cord transection. By 1990, Beattie and co-workers [19] had been able to characterize the response to spinal cord transection histologically and using subjective functional recovery observations in stage 49 to stage 62 animals. We have previously reported a decrease in functional recovery after spinal cord transection in animals from stages 50, 54, 58 and 66, observing a progressive decrease in regenerative ability as metamorphosis proceeded, using a qualitative method to evaluate recovery [21].

Here, we present a comparative analysis of axonal growth after spinal cord transection in stage 50 (R-stage) and stage 66 (NR-stage) using immunodetection of acetylated tubulin. We observed the first differences at the axonal growth level within the first 2 days after transection, where axons wrapped around the stumps in the R-stage but not in the NR-stage. By 6 days after transection, axons started to extend their tips into the lesion site, as previously described for the newt [32]. R-stage animals, therefore, show a response akin to newts, which can regenerate throughout their lifespan.

Therefore, the first histological differences between R- and NR-stages in their response to spinal cord transection were observed within the first few days after the injury. These early differences allowed us to select experimental time points for the transcriptome-wide profiling.

### The transcriptome deployed in response to spinal cord injury shows extensive differences between regenerative and non-regenerative stages

According to the early differences observed in the response to spinal cord injury in R- and NR-stages, we selected 1, 2, and 6 dpt for high-throughput RNA sequencing. Our experimental design allowed us to perform pairwise comparisons not only between sham and transected animals, but also between R- and NR-stages, thus allowing us to identify: (1) transcripts that responded to damage to the spinal cord only, leaving out damage to other tissues, and (2) those that showed a different response to injury when comparing these stages. The latter group is the key advantage to our experimental model, as it represents the differences in the response to injury between R- and NR-stages, and could therefore explain their differences in regenerative capability.A global picture of the results obtained revealed that the transcriptome in response to spinal cord transection displayed the following key differences when comparing the R-stage with the NR-stage. First, the number of differentially expressed transcripts was higher at 1 dpt in the R-stage, decreasing progressively towards 6 dpt, while the NR-stage showed the highest number of differentially expressed transcripts at 6 dpt (Figure 2b). Second, out of all differentially expressed transcripts detected in both stages, only 19% were regulated in both stages, while the remaining 81% were regulated in either stage (Figure 3a). Third, genes involved in neurogenesis and axonal regeneration, both categories directly related to spinal cord injury and regeneration, showed very different expression profiles when comparing R- and NR-stages. Finally, gene ontology analysis showed that most enriched biological processes regulated in each stage were either unique to each stage, or showed a different timing in their enrichment or the length of it (Figures 5, 6, 7). In addition, transcripts from categories regulated in both stages were not the same in R- and NR-stages, and we were able to validate their expression changes in biological replicates using RT-qPCR, further supporting the robustness of our results. Therefore, at a global level, we observed important differences in the timing of the transcriptional response, and in the repertoire of regulated transcripts and biological processes after spinal cord injury when comparing R- and NR-stages.

### Differential regulation of transcripts from gene ontologies directly related to spinal cord regeneration

As mentioned previously, key differences between the responses to injury in R- and NR-stages included the gene ontologies ‘neurogenesis’ and ‘axonal growth cone’, predicted to be related to spinal cord regeneration. The cellular and molecular differences between regenerative and non-regenerative organisms, which allow amphibians and teleost fish to regenerate, or those inhibiting regeneration in mammals, remain unknown. However, neurogenesis and axonal regeneration have been proposed to contribute to the regenerative process after a massive loss of neurons and glia due to injury, in addition to the interruption of axonal tracts [6,7].

Constitutive neurogenesis occurs in the spinal cord of *Xenopus* R-stages [49]. After spinal cord hemisection in stage 56 animals, there is regeneration of supraspinal axonal tracts, including reticular nuclei [50]. In mammals, however, although ependymal cells have been shown to have neural stem or progenitor activity after injury, they only give rise to astrocytes and oligodendrocytes, but not to new neurons [10]. There is no evidence for axonal regeneration in the adult mammalian central nervous system [51].

We found that in the ‘neurogenesis’ category, transcripts for *neurod4*, *MGC83023*, and *ascl1* were exclusively upregulated in the R-stage (Figure 4a). *Neurod4* (*Neurogenic differentiation 4*) and *ascl1* (*Achaete-scute complex homolog 1*) are both proneural transcription factors [52,53], and while *MGC83023* has not been characterized previously, it has a 97.3% sequence identity to the *Xenopus tropicalis* transcript for *achaete-scute homolog 1-like*. This specific upregulation in the R-stage of proneural transcription factors suggests a differential regulation of the neurogenic process during regeneration. Nevertheless, although we expected a similar specific upregulation of more transcripts from this category, a group of them showed an early upregulation in the R-stage at 1 and 2 dpt, and a delayed upregulation in the NR-stage at 6 dpt (Figure 4, II). Upregulation of these transcripts supports that neurogenesis could be taking place after injury in the R-stage, and that timely activation of the neurogenic program is required for successful spinal cord regeneration.

Another gene ontology category regulated differentially included transcripts that belong to the axonal growth cone group. More than half of these transcripts showed a strong downregulation at 6 dpt in the NR-stage, which was not observed in the R-stage. We found several factors that could explain failure of axonal growth cone extension. For example, external cues like semaphorins and *reelin*, and receptors that respond to extracellular cues like *EPH receptors*, *ngfr(p75)* and the *netrin-1* receptor *dcc* (Figure 4, III, and Table 4). The latter has been shown to mediate a turning response in retinal ganglion cell growth cones in *Xenopus*[54]. Conversely, the intracellular response machinery to extracellular cues was also altered, including downregulation of the *cdk5r1* activator of CDK5 and microtubule-associated proteins. All of these molecules were mainly deregulated in the NR-stage and are in agreement with the degeneration of the distal part of severed axons and the lack of neurite plasticity events, such as sprouting or axonal regeneration [13], which could, in part, explain the lack of functional recovery in NR-stage animals.

Therefore, transcripts related to neurogenesis and axonal regeneration were differentially regulated, with specific upregulation of three proneural transcription factors in the R-stage and considerable downregulation of growth cone transcripts and deregulation of axonal guidance cues in the NR-stage. Furthermore, the fact that these known processes were regulated differentially in R- and NR-stages supports this comparative experimental paradigm in *Xenopus laevis* as a model to identify the molecular mechanisms that allow regeneration in the R-stage or inhibit it in the NR-stage.

### Different biological processes were regulated in regenerative and non-regenerative stages

Another key difference in the response to spinal cord transection was observed at a global level in the gene ontology enrichment analysis. Differentially expressed transcripts in R- and NR-stages had different global profiles of enriched processes, and they could be arranged into two main groups: (1) processes related to stem or progenitor cell maintenance and differentiation, and (2) processes involved in providing an either permissive or non-permissive environment for regeneration.

In the first group, processes related to stem or progenitor cell maintenance and differentiation, we identified the following biological processes: metabolic processes, cell cycle and developmental processes. We found that a remarkable predominance of metabolic processes were enriched in the R-stage (Figures 5, 6; purple), and seven out of ten top regulated transcripts belonged to genes involved in metabolism. This suggests a high regulation of metabolic processes after spinal cord injury in the R-stage. Recently, there have been several reports on stem cell metabolism and the role this plays in pluripotency maintenance [55-58]. These associate a highly glycolytic metabolism with ‘stemness’ and cell proliferation, whereas the switch towards oxidative metabolism causes a shift towards differentiation. Furthermore, recent work from the Daley group showed that Lin28 enhances tissue repair through reprogramming of cellular metabolism in different injury models in mice [59]. The predominance in our results of transcripts related to metabolic processes regulated after injury in the R-stage supports the notion of metabolism as a key regulator of endogenous stem cells and their capacity to differentiate during neurogenesis.In addition, enrichment of upregulated cell cycle genes at 1 and 2 dpt in the R-stage (Figure 5, blue) and a concomitant transient (only 1 and 2 dpt) downregulation of developmental genes (Figure 6, orange) suggests a stem cell proliferation phase followed by a differentiation phase in which cell proliferation ceases, developmental processes are no longer repressed anymore, allowing differentiation.

Processes involved in promoting a permissive or non-permissive environment for regeneration were the immune response and inflammation, oxidation and reduction, and response to stress. The main differences between R- and NR-stages were that amongst upregulated transcripts, immune response and inflammation was only enriched in the NR-stage (Figure 5, green); amongst downregulated transcripts oxidation and reduction was only enriched in the R-stage (Figure 6, yellow); while the response to stress was enriched transiently (1 and 2 dpt) in the R-stage (Figure 5, pink), but enriched at all time points in the NR-stage (Figures 5, 6, pink). It has been proposed that the mammalian spinal cord provides a non-permissive environment for neurogenesis because neural stem and progenitor cells (NSPCs) are present (ependymal cells), but they only give rise to glial cells, not to new neurons [10]. However, when spinal cord NSPCs isolated by stimulation with FGF2 in mammals are transplanted into a neurogenic niche in the brain (for example, dentate gyrus), they give rise to new neurons. This raises the question of which are the extrinsic cues that are permissive for neuronal differentiation, and which are non-permissive, like those present in the mammalian spinal cord. Regarding this, while both positive and negative effects have been associated with the immune and inflammatory responses after spinal cord injury in mammals, regenerative models have been shown to have a less developed or more controlled response [60-63].

The immune response is also associated with oxidation-reduction processes, as leukocytes can have iNOS activity and therefore be involved in the production of reactive oxygen species. A higher infiltration of iNOS positive leukocytes in metamorphic stage *Xenopus* tadpoles (described as non-regenerative in this study) has been correlated to the lack of tail regeneration [63]. The enrichment pattern for immune response and inflammation and for oxidation-reduction processes in our results support this evidence. Finally, a sustained response to stress could also be associated with the maintenance of a non-regenerative permissive environment.

While neurogenesis and NSPC activity, and the presence of either permissive or non-permissive environments had been proposed to be key factors during spinal cord regeneration, our results shed light on which factors could be regulating these processes. The results obtained here not only suggest metabolic, developmental and proliferation processes as differentially regulated in response to injury, but also provide an integrative view of how they could interact during spinal cord regeneration, and which genes in particular are being regulated. The same applies to the role of the immune response and inflammation, response to stress and oxidation-reduction and their effect as extrinsic factors affecting regeneration. This is valuable information as it is this knowledge of how processes predicted to contribute to spinal cord regeneration can be modulated that will finally provide new strategies to promote spinal cord regeneration in mammals.

## Conclusions

We have obtained the first genome-wide expression profile of the response to spinal cord injury comparing R- and NR-stages in *Xenopus laevis*. We identified extensive differences in the responses deployed by these two stages. The most important were: (i) R-stage tadpoles deployed extensive transcriptional changes at 1 day after injury, while in NR-stage froglets, we observed this only by 6 days after injury; (ii) R- and NR-stages regulated a very different set of transcripts, including previously uncharacterized transcripts, with no more than 20% at all time points being regulated in both stages; (iii) we detected differential regulation of transcripts involved in different biological processes, including neurogenesis and axonal regeneration, metabolism, immune response and inflammation, cell cycle, development and response to stress, and validated this differential regulation using RT-qPCR.

The genes and biological processes that we have identified contribute to a better understanding of the genetic mechanisms of spinal cord regeneration, and will enable the design of future approaches to promote regeneration in animals that cannot carry out this process. Furthermore, these results introduce this regenerative versus non-regenerative comparative experimental paradigm in *Xenopus laevis* as a genetic tool to identify new mechanisms of spinal cord regeneration. Using this model system’s unique advantage of R- and NR-stages, which can both be readily manipulated and evaluated, we can continue to generate new knowledge on spinal cord regeneration, which will provide the basis for new strategies to promote spinal cord regeneration in mammals.

## Methods

### Growth of *Xenopus laevis* tadpoles and froglets

Animals were obtained by *in vitro* fertilization and cultured as described [21] using frogs from Nasco (USA), until they achieved Nieuwkoop and Faber stages 49 to 51 for the regenerative stage (R-stage), and stage 66 for the non-regenerative stage (NR-stage). All animal procedures were approved by the Comision de Bioetica y Bioseguridad from the Faculty of Biological Sciences, Pontificia Universidad Católica de Chile.

### Spinal cord transection

Animals were anesthetized in 0.01% MS222 (ethyl 3-aminobenzoate methanesulfonate) prior to surgery. Iridectomy scissors were used to make a dorsal incision of skin and muscle at the midpoint between the fore and hind limbs (mid-thoracic, approximately between the seventh and eighth vertebrae), and limb buds were used as a reference in the R-stage. The spinal cord was then cross-sectioned (transected), interrupting all ascending and descending tracts. For sham operation, the same dorsal incision was performed, without damaging the spinal cord. Animals were then transferred to tanks containing 0.1× Barth (8.9 mM NaCl; 102 μM KCl; 238.1 μM NaHCO_3_; 1 mM 4-(2-hydroxyethyl)-1-piperazine-ethane sulfonic acid (HEPES); 81.14 μM MgSO_4_; 33.88 μM Ca(NO_3_)_2_; 40.81 μM CaCl_2_, pH 7.6) supplemented with antibiotics (100 μg/ml penicillin and 100 μg/ml streptomycin), at a density of one animal per 35 to 50 ml for both stages. Tanks were aerated, and animals were fed every other day, starting 24 hours after operating. Siblings were used for all transected and sham pairs, to minimize variation.

### Immunofluorescence

Animals were sacrificed at selected time points and fixed in 4% paraformaldehyde for 2 hours at room temperature or overnight at 4°C. For immunofluorescence, samples were cryoprotected in 20% sucrose and embedded in optimal cutting temperature compound. Samples were then frozen and sectioned at 10 μm. Mouse monoclonal anti-acetylated α-tubulin (1:1,000, Sigma T7451) was used to detect axonal tracts, with AlexaFluor® 488 (1:500) as a secondary antibody. Nuclei were stained using TOTO3 (1:1,000, Molecular Probes T3604).

### Spinal cord isolation

To isolate spinal cords, R- and NR-stage transected or sham-operated animals were sacrificed at 1, 2 or 6 days post transection (dpt) or post sham operation (dps), and a caudal spinal cord segment was dissected. For the R-stage, this segment went from the transection site to the midpoint between the hindlimb bud and the tip of the tail, was 9 mm long, and had an approximate diameter of 0.3 mm at its widest, narrowing strongly towards the tip of the tail. For the NR-stage we isolated the segment from the transection site to the end of the spinal cord, which was 3 mm long and had an approximate diameter of 0.7 mm. Twelve single replicate samples were prepared: six for the R-stage (1 dps, 1 dpt, 2 dps, 2 dpt, 6 dps, 6 dpt), each sample prepared from pools of 35 to 50 tadpole spinal cords, and six for the NR-stage (1 dps, 1 dpt, 2 dps, 2 dpt, 6 dps, 6 dpt), each sample prepared from pools of 5 or 6 froglet spinal cords.

### Library preparation and high-throughput sequencing

The RNA-Seq samples and sequencing libraries were prepared as described [64]. Briefly, dissected spinal cords were placed immediately in at least ten volumes of RNAlater solution (QIAGEN) to maintain RNA integrity in the sample. Total RNA was isolated using the RNeasy Mini Kit (QIAGEN) according to the manufacturer’s instructions, and eluted in water. DNase I (QIAGEN) treatment was included in the protocol to avoid genomic DNA contamination. RNA concentration was measured using Nanodrop (Thermo Scientific), and the integrity was determined using the 2100 Bioanalyzer (Agilent Biotechnologies), ensuring that for all cases the RNA integrity number was higher than 8. Library preparation and high-throughput sequencing were outsourced to Macrogen Inc. Twelve RNA-Seq libraries were prepared from 1 μg total RNA using the TruSeq RNA Sample Prep Kit (Illumina). This kit allows the isolation of the polyadenylated fraction and performs fragmentation before reverse transcription. The library size was approximately 400 bp for all samples. Paired-end 100 bp sequencing was performed using the HiSeq 2000 (Illumina), yielding an average of 10 GB data per sample (approximately 80 million reads).

### Read processing and mapping

A quality check was performed for all libraries using the FastQC (version 0.10.0) software. Each pair from the paired-end sequencing was examined separately. The first 14 and last 27 nucleotides (nt) were trimmed from all reads because we detected a decrease in sequencing quality, leaving 60 nt reads. This process was performed simultaneously with the next step (mapping to reference) using Bowtie 1 (v0.12.8), with which reads were trimmed and mapped in pairs to the *Xenopus laevis* transcript reference from UniGene (Build #90) [65]. Bowtie flags were ‘--trim5 14’ for trimming the first 14 bases, d ‘--trim3 27’ for trimming the last 27, ‘-a’ for reporting all alignments per read, ‘-v 2’ for allowing up to two mismatches in the alignment, ‘-p 8’ for aligning using eight threads to increase mapping speed, ‘-m 1’ for reporting only alignments that had one mapping position in the reference, and ‘-I 200’ and ‘-X 300’ for the minimum and maximum distance between pairs of reads in the alignments, except for 1 dpt and 1 dps samples from R- and NR-stages, where ‘-I 300’ and ‘-X 400’ were used.

### Analysis of differentially expressed transcripts in response to spinal cord transection

Differentially expressed transcripts in response to spinal cord transection were determined using edgeR (version 2.2.5) [66], which allowed us to calculate the trimmed mean of *M* values [67]. This permitted us to (1) normalize the level of each transcript to the total number of reads detected in each sample, and (2) determine which transcripts showed significantly different levels when comparing transected and sham-operated controls. This yielded six lists (R1, R2, R6, NR1, NR2, and NR6), containing the following information for each mapped transcript: (i) FC corresponding to log_2_(transected/sham); (ii) total abundance corresponding to log_2_(transected + sham); (iii) *P* value; and (iv) read counts observed in each transected and sham-operated control samples (see Additional file 6). To select transcripts with a significant differential expression, the following criteria were used: (i) FC ≥ 2; (ii) *P* < 0.01, and (iii) sum of reads in transected and sham > 10.

*MA* plots were generated by plotting the *M* value on the *y*-axis, corresponding to the log_2_(transected/sham) ratio, and the *A* value on the *x*-axis, corresponding to the total abundance log_2_(transected + sham). Transcripts that met differential expression criteria were marked with blue dots for upregulated transcripts and orange dots for downregulated transcripts, for better visualization.

### Identification of transcripts that showed a different response to transection between regenerative and non-regenerative stages

The differentially expressed transcripts contained in lists R1, R2, R6, NR1, NR2, and NR6 were further selected to obtain those that responded differently to spinal cord injury in R- and NR-stages. For this a pairwise comparison of those lists was performed (R1 versus NR1; R2 versus NR2; R6 versus NR6). An FC ratio was defined as the ratio between the FC of a given transcript in one stage, divided by the FC of the same transcript in the other stage. The ‘FC ratio’ is a measure of how different is the response to spinal cord injury between the R- and NR-stages. Transcripts with an FC ratio ≥ 2 or ≤ 0.5 were considered to respond differently between the stages studied. This definition includes transcripts with the following expression patterns in R- and NR-stages: (i) transcripts with a completely opposite response in R- and NR-stages (for example, upregulated in R-stage and downregulated in NR-stage); (ii) transcripts that were differentially expressed in one stage and had no change or were absent in the other stage; (iii) transcripts with differential expression in both stages and in the same direction but with a difference in the magnitude of the change that was larger than two-fold (for example, three-fold upregulation in R-stage and eight-fold upregulation in NR-stage).

The top differentially regulated transcripts were selected using a higher filter for the number of reads, using a minimum abundance ≥ mean value of the sum of reads (transected + sham) from both stages in each list, and sorted according to the FC ratio.

### Transcript annotation

*Xenopus laevis* transcript annotation was downloaded from UniProtKB [68] (release 2012_07). We used only entries that could be assigned to a UniGene cluster and had a gene ontology annotation. As we had detected differentially expressed transcripts that did not have any assigned GO term in UniProtKB, we annotated them using Blast2GO [69] (version 2.6.6, database b2g_aug12). A BLAST [70] search was performed against the non-redundant database of proteins from the NCBI (NR version 4, 8 November 2012), using the algorithm blastx of the blastall command (version 2.2.18). Non-significant alignments were filtered out (‘-e 0.001’) and only the first 20 alignments were used for annotation. The flag ‘-a 8’ was used to speed up the alignment process. The Blast2GO default settings were used. Also, InterPro [71] results obtained within the software were merged to the annotation. Finally, the annex function was run on the annotation.

### Gene ontology enrichment

The Blast2GO tool was used to perform GO enrichment for transcripts that showed a different response to transection in R- and NR-stages. We used the annotation of all known *Xenopus laevis* proteins in the UniProt Knowledgebase database as a background for the contingency table of the Fisher’s exact test. Transcript lists from each day and stage were separated into up- and downregulated and were analyzed separately. Overrepresented GO terms with a false discovery rate (FDR) < 0.05 were reported.

Slimming was performed for GO terms in the biological process category to identify the most representative processes. Briefly, the level of each GO term was obtained using Blast2GO by making a combined graph (Analysis → make combined graph; filter used, minimum), filtering out terms that contained less than 10% of the total number of selected transcripts in the list. The Blast2GO tool outputs a table used to build a directed acyclic graph depicting each GO term in its corresponding level. Enriched terms in the third level were selected from the table, and pie charts were constructed using the number of transcripts in each category. In addition, categories were manually classified into the following groups: cell cycle, response to stress, developmental processes, metabolic processes, immune response and inflammation, cell death, and oxidation-reduction.

### Heat map generation and clustering

Transcripts associated with biological processes that contained the words ‘neurogenesis’ or ‘growth cone’ were found to construct two lists containing transcripts related to these two processes. Transcripts were hierarchically clustered according to their logFC value using Cluster 3.0 [72]. The similarity metric used was ‘correlation (centered)’, and the clustering method used was ‘average linkage’. Output .cdt files were opened in Java TreeView [73] for graphic representation of clustering results.

### RT-qPCR for differential transcript expression validation

Two independent biological replicates, using the same experimental conditions described previously, were prepared from 10 to 15 animals for the R-stage, and 2 to 3 for the NR-stage. Total RNA was obtained as described previously, to give a total of three biological replicates (including the sample prepared for RNA sequencing). The cDNA was synthesized using the M-MLV reverse transcriptase (Promega), and qPCR was performed using Power SYBR Green (Applied Biosystems) or Maxima SYBR Green (Thermo Scientific) by performing three technical replicates on at least two independent biological replicates. The relative expression ratio was then calculated as described [74], using *eef1a1* (GenBank: BC043843) as a reference gene. Primer sequences are available upon request.

## Abbreviations

dps: days post sham operation; dpt: days post transection; FC: fold change; FC ratio: ratio between R- and NR-stage fold changes; HEPES: 4-(2-hydroxyethyl)-1-piperazine-ethane sulfonic acid; MS222: ethyl 3-aminobenzoate methanesulfonate; NR: non-regenerative; NSPC: neural stem or progenitor cell; R: regenerative; RT-qPCR: quantitative RT-qPCR; reverse transcriptase quantitative polymerase chain reaction; RNA-Seq: high-throughput RNA sequencing.

## Competing interests

The authors declare that they have no competing interests.

## Authors’ contributions

DLL performed most experiments. MM participated in RNA-Seq sample preparation and RT-qPCR validation. LIA and FM carried out most bioinformatics analyses. VST and JvM helped in RT-qPCR validation. RM performed immunofluorescence experiments. MG helped in the design of the study and sample preparation. DLL and JL designed experiments, performed data analysis, and wrote the manuscript. All authors read and approved the final manuscript.

## Supplementary Material

Additional file 1 **Quality of RNA-Seq results for regenerative and non-regenerative stage samples.** Total reads: number of total reads obtained per sample. Mapped reads: number of reads that mapped to the reference (*Xenopus laevis* UniGene mRNA database). Mapped transcripts: total number of transcripts to which at least one read was successfully mapped. Q30 or higher (%): percentage of reads that had an average sequencing quality of Q30 or higher. R1, R2, R6: regenerative stage at 1, 2, or 6 days post-operation (respectively). NR1, NR2, NR6: non-regenerative stage at 1, 2, or 6 days post-operation (respectively). Click here for file

Additional file 2 **Transcripts with a significant differential expression in response to spinal cord transection.** Transcripts meeting differential expression criteria (significant fold change (FC), see Methods and Figure 2a) when comparing transected and sham-operated samples are shown, ordered by descending FC value. Each sheet corresponds to data from either regenerative (R) or non-regenerative (NR) stages, at 1, 2 and 6 days after injury. log2Conc, absolute transcript abundance in a log2-scale; log2FC, fold change in a log2-scale. NA refers to transcripts that were not detected in the sample. R1, R2, R6: regenerative stage at 1, 2, and 6 days post-operation (respectively). NR1, NR2, NR6: non-regenerative stage at 1, 2, and 6 days post-operation (respectively). Click here for file

Additional file 3 **Transcripts that show a different response to spinal cord injury in R- and NR- and stages.** Transcripts that respond differently in regenerative and non-regenerative stages with an FC ratio ≥ 2 or ≤ 0.5 are shown, ordered by descending FC ratio. Total reads: sum of all reads detected in sham and transected samples for each specific day in regenerative and non-regenerative stages. log2Conc, absolute transcript abundance in a log2-scale; log2FC, fold change in a log2-scale. NA refers to transcripts that were not detected in the sample. R1, R2, R6: regenerative stage at 1, 2, and 6 days post-operation (respectively). NR1, NR2, NR6: non-regenerative stage at 1, 2, and 6 days post-operation (respectively). Click here for file

Additional file 4 **Gene ontology enrichment analysis for differentially expressed transcripts 1 day after injury.** Biological processes enriched amongst up or downregulated transcripts at 1 day after injury are shown. A list of genes contributing to each category is shown. Transcribed loci that have not been characterized in *Xenopus* before are shown as ‘unknown’. FDR, false discovery rate; #Test, number of transcripts contributing to each category. Click here for file

Additional file 5 **Gene ontology enrichment analysis for differentially expressed transcripts 2 days after injury.** Biological processes enriched amongst up or downregulated transcripts at 2 days after injury are shown. A list of genes contributing to each category is shown. Transcribed loci that have not been characterized in *Xenopus* before are shown as ‘unknown’. FDR, false discovery rate; #Test, number of transcripts contributing to each category. Click here for file

Additional file 6 **Gene ontology enrichment analysis for differentially expressed transcripts 6 days after injury.** A list of genes contributing to each category is shown. Transcribed loci that have not been characterized in *Xenopus* before are shown as ‘unknown’. FDR, false discovery rate; #Test, number of transcripts contributing to each category. Click here for file
